# Puerarin: A protective drug against ischemia-reperfusion injury

**DOI:** 10.3389/fphar.2022.927611

**Published:** 2022-08-24

**Authors:** Minglang Gao, Ziyao Zhang, Kai Lai, Yu Deng, Chuanbing Zhao, Zilong Lu, Qing Geng

**Affiliations:** ^1^ Department of Thoracic Surgery, Renmin Hospital of Wuhan University, Wuhan, China; ^2^ Department of Pancreatic Surgery, Renmin Hospital of Wuhan University, Wuhan, China

**Keywords:** puerarin, ischemia-reperfusion injury (I/R), myocardium, brain, lung, spinal cord, intestine

## Abstract

Ischemia-reperfusion (I/R) is a pathological process that occurs in numerous organs throughout the human body and is frequently associated with severe cellular damage and death. Puerarin is an isoflavone compound extracted from the root of *Pueraria* lobata and has pharmacological effects such as dilating cerebral vessels and anti-free radical generation in cerebral ischemic tissues. With the deepening of experimental research and clinical research on puerarin, it has been found that puerarin has a protective effect on ischemia-reperfusion injury (IRI) of the heart, brain, spinal cord, lung, intestine and other organs. In summary, puerarin has a vast range of pharmacological effects and significant protective effects, and it also has obvious advantages in the clinical protection of patients with organ IRI. With the deepening of experimental pharmacological research and clinical research, it is expected to be an effective drug for IRI treatment. In this review, we summarize the current knowledge of the protective effect of puerarin on I/R organ injury and its possible underlying molecular mechanisms.

## Introduction

Ischemia-reperfusion injury (IRI) is a pathophysiological process that can occur in various organs such as the heart, brain, lung, and intestine, resulting in a variety of clinical diseases. IRI is a severe pathological condition that poses a significant therapeutic challenge for doctors (Wu et al., 2018; Yan et al., 2020) and IRI is a critical condition for which doctors must control cell damage and preserve organ function (Wu et al., 2018).

**FIGURE 1:**
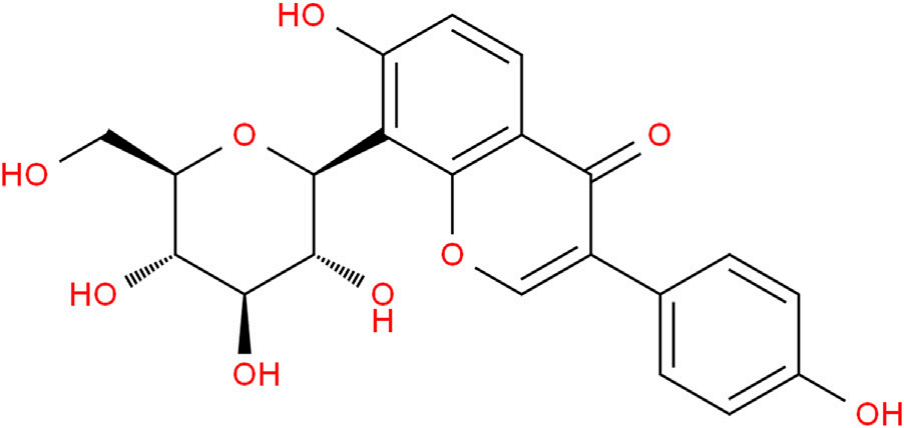
The chemical structure of puerarin.

Gegen is a kind of Chinese medicine used for various medicinal purposes. The flavonoid content of it is high, and the most abundant flavonoid is puerarin (Guerra et al., 2000). As a naturally occurring isoflavone, puerarin (Figure 1) is frequently used to treat cardiovascular symptoms in China (Feng et al., 2010). It has pharmacological effects such as improving cardiovascular and cerebrovascular circulation, reducing myocardial apoptosis, anti-oxidative stress, dilating cerebral vessels, and improving vascular endothelial function. It is also clinically used for the treatment of cardiac and vascular diseases. Puerarin is isolated from Gegen and has been widely studied and applied to various illnesses since the 1950s (Zhang et al., 2019).

**FIGURE 2:**
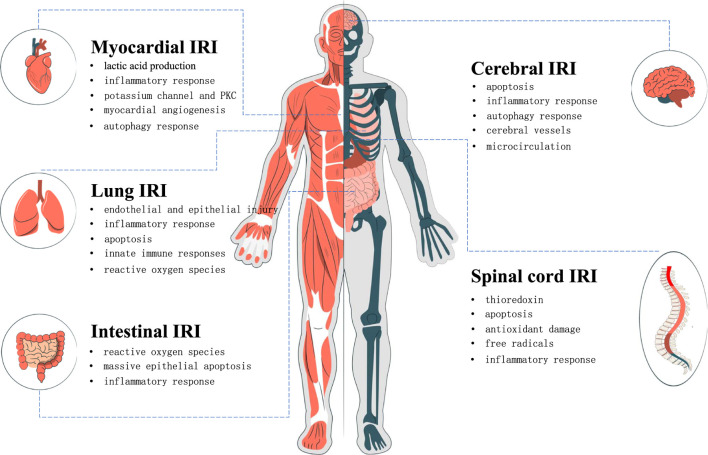
The protective effect of puerarin on human organs. Protective mechanism of puerarin in ischemic reperfusion injury of heart, brain, lung, spinal cord and intestine.

In recent years, it has been found that puerarin has an excellent protective effect on the injury of the heart, brain, lung, spinal cord, intestine, and other organs after I/R (Figure 2). Due to its wide range of pharmacological effects, puerarin can protect organs from IRI through various mechanisms, such as reducing lactic acid production, inhibiting inflammatory responses, antioxidants, promoting angiogenesis, and inhibiting autophagy responses, and so on (Zhou F. et al., 2014). The mechanism is summarized as follows.

## Protective effect on myocardial ischemia

Acute myocardial infarction (AMI) can lead to irreversible damage and necrosis of myocardial cells. IRI is one of the main problems in treating myocardial infarction (Yellon and Baxter, 2000; Lunder et al., 2012; Szczesny et al., 2014; Long et al., 2015; Yılmaz et al., 2015). Apoptosis is one of the essential mechanisms of myocardial ischemia-reperfusion injury (MIRI), and it is also an important factor in the development of myocardial damage and heart failure. Experimental studies have shown that puerarin can reduce myocardial apoptosis by reduction of lactic acid production, inhibition of inflammatory response, inhibiting oxidative stress, opening the potassium channel and activating protein kinase C (PKC), promoting myocardial angiogenesis and inhibiting autophagy, exerting a protective effect on MIRI (Fan et al., 1992a; Fan et al., 1992b; Zhu et al., 2001; Xie et al., 2003; Gao et al., 2005; Yang et al., 2005; Gao et al., 2006; Pan et al., 2006; Wu et al., 2006; Zhang et al., 2006; Gao et al., 2007; Ai et al., 2015; Ma et al., 2016; Tang et al., 2017; Boengler et al., 2018; Mishra et al., 2019; Xu et al., 2019; Davidson et al., 2020; Wang et al., 2020; Zhao et al., 2021).

In the past 2 decades, it has been recognized that apoptosis is the primary modality of myocardial cell death in IRI (Mishra et al., 2019; Davidson et al., 2020). Cardiomyocyte apoptosis occurs through an endogenous pathway in response to DNA damage, reactive oxygen species (ROS), and increased intracellular Ca2+ levels. Apoptosis requires energy and involves the release of mitochondrial cytochrome C and activation of cysteine aspartate proteases, leading to regular DNA breaks (Mishra et al., 2019). Mitochondria are the primary targets of all myocardial protective signaling pathways under ischemia, and they are also the key to initiating necrosis and apoptosis when mitochondria become dysfunctional during I/R (Boengler et al., 2018).

### Reduction of lactic acid production

Previous studies revealed that puerarin could reduce lactic acid production to protect the heart from myocardial reperfusion injury after cardiac arrest. Fan studies on myocardial metabolism and ultrastructure were made. Oxygen consumption, lactate production, Creatine Kinase (C.K.) release, water content and ultrastructural changes during myocardial reperfusion were measured. The results showed that intermittent infusion of a cardioplegic solution containing puerarin significantly decreased myocardial lactate production during ischemia and myocardial oxygen consumption, C.K. release, and water content during reperfusion (Fan et al., 1992a). Under electron microscopy, the degree of ischemic damage judged by a scoring method was less pronounced in the puerarin group than in the control group (Fan et al., 1992b). These suggest that puerarin has protective effects on the function of hearts that had undergone long periods of arrest and reperfusion, possibly in part by reducing lactic acid and oxygen consumption (Fan et al., 1992a; Fan et al., 1992b).

### Inhibition of inflammatory response

To investigate the mechanism further, Zhu found that the activities of Tumor Necrosis Factor α (TNF-α) and interleukin 6 (IL-6) secreted by myocytes are enhanced by the stimulation of hypoxia and reoxygenation; puerarin inhibits TNF-α during hypoxia, while lower doses of puerarin augmented it during reoxygenation; puerarin can inhibit the over secretion of IL-6 in a dose-dependent manner (Zhu et al., 2001). A similar Liu’s study then demonstrated that puerarin inhibits inflammatory responses, reduces neutrophil infiltration and oxidative stress in the heart, and protects against AMI induced by severe burn (Liu et al., 2015). while puerarin might have a protective effect in myocardial tissues during MIRI through increasing superoxide dismutase (SOD) and decreasing C.K. and methylenedioxyamphetamine (MDA) (Wenjun et al., 2015). Puerarin may protect against MIRI by inhibiting inflammatory responses, probably via the SIRT1/nuclear factor κB (NF-κB) pathway, and inhibiting the NOD-like receptor thermal protein domain associated protein 3 (NLRP3) inflammasome is also involved in puerarin-induced cardioprotective effects (Wang et al., 2020). In conclusion, puerarin may inhibit the inflammatory response by inhibiting the secretion of inflammatory factors and reducing the activation of inflammatory pathways, thereby protecting the heart from ischemia-reperfusion injury.

### Opening the potassium channel and activating PKC

Xie studied the protective effect of puerarin injection on MIRI in patients with angina pectoris and its mechanism, which may be related to its effect in lowering plasma vWF: Ag and ET-1 and increasing the serum NO content (Xie et al., 2003).The study by Gao and Pan proved that puerarin protects the myocardium against IRI via inhibiting mitochondrial permeability transition pore opening and activating the mitochondrial ATP-sensitive potassium channel or opening the calcium-activated potassium channel and activating PKC, while 5-hydroxydecanoate attenuated the effects of puerarin (Gao et al., 2005; Gao et al., 2006; Pan et al., 2006; Gao et al., 2007). After a while, Tang and his team experimentally verified the protection of puerarin in cardiomyocytes from anoxia/reoxygenation injury is mediated by PKC(Tang et al., 2014).

### Promoting myocardial angiogenesis

Puerarin can also induce the expression or activation of angiogenesis-related genes, such as vascular endothelial growth factor (VEGF), hypoxia-inducible factor 1 α (HIF-1 α) and endothelial nitric oxide synthase (eNOS). A previous study in rats showed that puerarin treated myocardial infarction by up-regulating the expression of VEGF and eNOS and promoting myocardial angiogenesis (Zhang et al., 2006). A similar study by Ai demonstrated that puerarin could accelerate cardiac angiogenesis and improve cardiac function of myocardial infarction rats by up-regulating VEGF (Ai et al., 2015). There are few studies on the gene expression of puerarin promoting angiogenesis, but its promotion of myocardial angiogenesis is reliable.

### Inhibition of autophagy response

**FIGURE 3:**
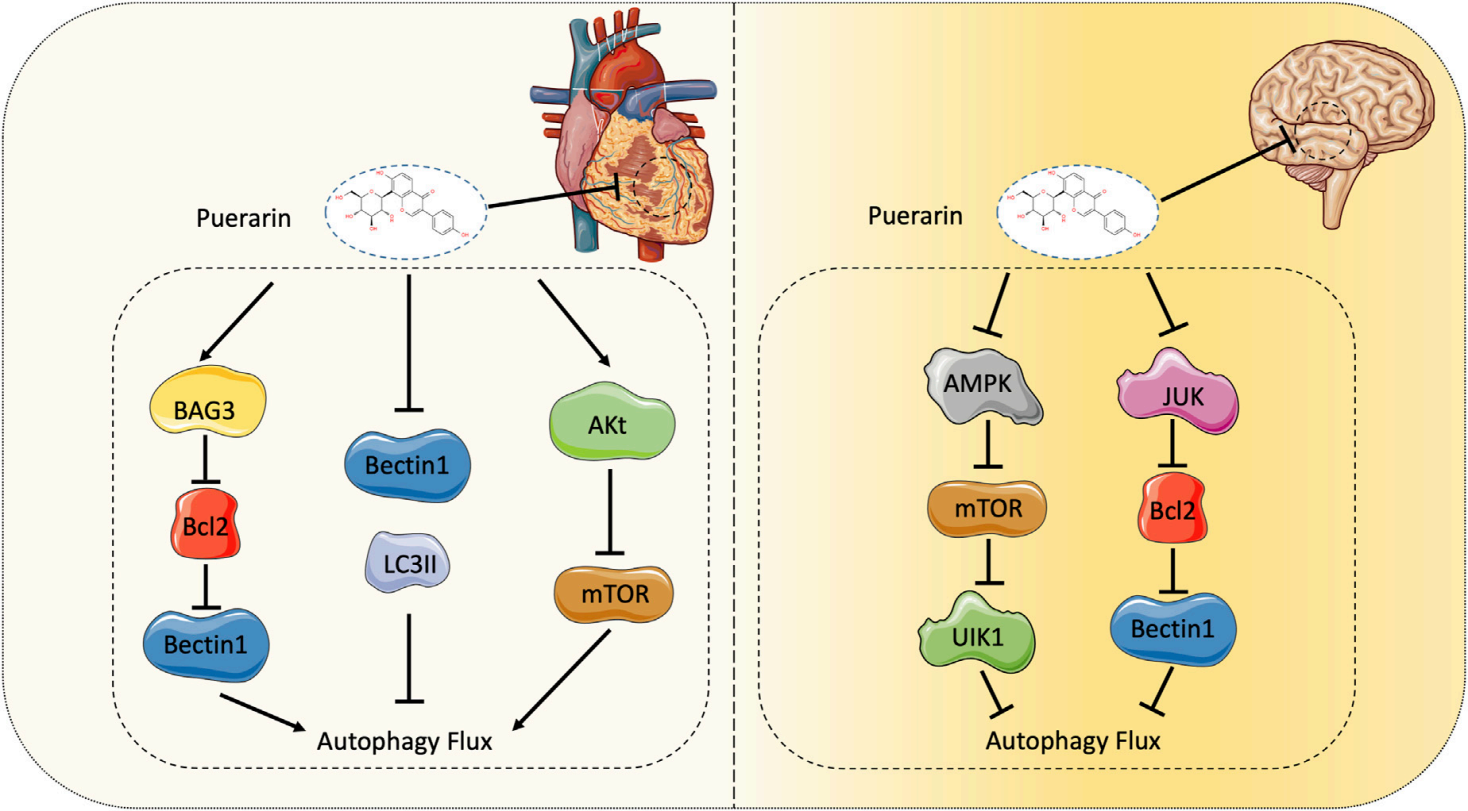
Peauarin protects myocardial and brain IRI *via* regulating autophagy.

Autophagy is an intracellular protective mechanism that is regulated by multiple proteins of autophagy-related genes (Atg), including the mammalian yeast Atg6 homolog Beclin-1 and the Atg8 homolog microtubule, the expression levels of microtubule associated light chain 3 (LC3), Beclin-1 and LC3β can reflect the level of autophagy. In the process of MI/RI, autophagy is activated due to changes in the external environment, and the signaling pathways that regulate autophagy in different periods are also different. Current studies have shown that during myocardial ischemia, due to reduced ATP production, Adenosine 5′-monophosphate (AMP)-activated protein kinase (AMPK) is activated, resulting in mTOR inactivation, thereby catalyzing UNC-51-like kinase 1 (ULK1)-induced autophagy. Studies have also confirmed that AMPK can directly activate ULK1 (Kim et al., 2011). After ULK1 is activated, the ULK1 complex is formed, which triggers the level III PI3K complex, and the interaction between the two forms a phagocytic vesicle, which expands to form an autophagosome. Fusion of autophagosomes to lysosomes is mediated by SNAREs, GTPase Rab7, and lysosome-associated membrane proteins (Mao et al., 2019). After the substrate is degraded, it can be released into the cytoplasm for recycling. During the reperfusion phase, because the cardiomyocytes are no longer ischemia and hypoxia, the AMPK pathway is inhibited and replaced by the Beclin-1 reperfusion phase. The specific mechanism may be related to the B-cell lymphoma-2 (Bcl-2) protein (Figure 3).

Puerarin significantly promoted Bcl-2 associated athanogene 3 (BAG3) expression in the rat primary cardiomyocytes after anoxia/reoxygenation injury (A/RI). BAG3 expression significantly stimulated autophagy in cardiomyocytes after A/RI, demonstrating protective effects on A/RI in cell viability and apoptosis (Ma et al., 2016). Puerarin pre-treatment may also attenuate myocardial IRI by inhibiting autophagy via the Akt signaling pathway (Tang et al., 2017). In the process of MI/RI, puerarin can also down-regulate the expression levels of proteins Beclin-1, LC3II and cytochrome C, thereby reducing excessive autophagy and apoptosis. Puerarin inhibits autophagy, promotes myocardial cell activity, inhibits apoptosis, reduces LDH and MDA content, and alleviates myocardial injury by regulating autophagy related genes of myocardial cells (Han et al., 2021). The experiment by Xu et al. demonstrated that puerarin protects against myocardial H/R injury by inhibiting apoptosis and oxidative stress (Xu et al., 2019).

Autophagy plays an important role in myocardial ischemia-reperfusion and its effect is bidirectional. Under normal circumstances, autophagy plays an important role in maintaining normal heart function. However, in cardiac pathology, excessive or insufficient autophagy may damage the myocardium. Some scholars believe that the process of autophagy is the process of regulating the balance of Yin and Yang. Insufficient autophagy will lead to the accumulation of metabolites, and excessive autophagy will lead to autophagy apoptosis. Only when autophagy is moderate, it will play a protective effect on cells, which happens to coincide with the self-harmony of Yin and Yang in traditional Chinese medicine theory (TCM) theory. In recent years, the study found that puerarin inhibit autophagy both improve myocardial ischemia-reperfusion injury, also can by promoting autophagy protect myocardial cells, whether this difference and the building time, building style, damage degree or animal age differences on currently is still unknown, but puerarin can reduce the myocardial I/R injury by autophagy, no doubt. It points out the direction for follow-up study, the use of moderate pue regulation of autophagy in the treatment of myocardial ischemia reperfusion injury, give full play to the traditional Chinese medicine therapy targets, regulation and characteristics of adverse reactions to small, and further to explore the regulation of autophagy in MI/RI mechanism, selective inhibition or activation of autophagy reduce myocardial injury, will become a new kind of treatment.

Similar clinical studies have demonstrated that the stress of operation and anesthesia could induce myocardial injury in patients with hypertension, which can be prevented by puerarin medicated during the perioperative period (Wu et al., 2006). Puerarin also alleviated coronary heart disease (CHD) in rats by inhibiting inflammation (Wang et al., 2020). These results suggest that puerarin may be a novel candidate for the treatment or protection of cardiac IRI.

## Protective effect on cerebral ischemia

The brain is an essential organ of life, and the abnormality of cerebrovascular can lead to complex pathological conditions, so ischemic cerebrovascular disease has high morbidity and mortality (Lee et al., 2018). Cerebrovascular ischemia can lead to neuronal cell death and cerebral infarction, clinically manifested as ischemic stroke, but its pathological mechanism is still unclear, and there is no specific drug. (Lee et al., 2018; Shin et al., 2020). The pathological process of cerebral ischemia is complex, involving many mechanisms and targeting few drugs. Developing neuroprotective agents from traditional Chinese herbs is a promising way to treat cerebral ischemia (Wu et al., 2010). Puerarin can dilate cerebral vessels, reduce cerebrovascular resistance, improve microcirculation, reduce apoptosis, relieve inflammatory response, reduce autophagy, and protect against cerebral ischemic injury. The clinical efficacy of adjuvant therapy for a cerebrovascular disease is satisfactory and has specific clinical application potential. A large number of experimental studies have shown that puerarin has a particularly protective effect on brain neuronal injury induced by acute cerebral ischemia, and the mechanism is multi-faceted (Cao et al., 2003; Lou et al., 2004; Chen et al., 2005; Yang et al., 2006; Chang et al., 2009; Wu et al., 2009; Han et al., 2012; Liu et al., 2013; Zhou Y. X. et al., 2014; He et al., 2017; Tao et al., 2017; Ling et al., 2018; Wang et al., 2018; Cheng et al., 2019; Huang et al., 2019)

### Decreased apoptosis

Puerarin ameliorates ischemic injury by reducing apoptosis. Chao’s study shows that B-cell lymphoma-2 (Bcl-2) can inhibit cell apoptosis, while puerarin can reduce cell apoptosis by regulating Bcl-2, thus improving cerebral ischemic injury. They also indicated that the inhibition of puerarin to cell apoptosis after cerebral resuscitation is related to its impact on the decrease in protein expression of the apoptosis-promoting gene, Fas and P53 (Cao et al., 2003; Yang et al., 2006). Similarly, Chang et al. demonstrated that puerarin is a potent neuroprotective agent on MCAO-induced focal cerebral ischemia *in vivo* (Chang et al., 2009). This effect may be mediated by the inhibition of both HIF-1 α and TNF-α activation, followed by the inhibition of inflammatory responses, apoptosis formation and neutrophil activation, resulting in a reduction in the infarct volume in I/R brain injury (Chang et al., 2009). Wu discovered that puerarin could also improve the learning-memory ability after rats’ global cerebral ischemia and reperfusion. The protective mechanism might be related to inhibiting or delaying the cell apoptosis by up-regulating the expression of Bcl-2 after I/R (Wu et al., 2009).

On the other hand, recent studies have explored the underlying mechanisms further. Han believed that puerarin reduced the apoptosis of neurocytes and had protective effects against cerebral IRI, possibly through activating the phosphatidylinositol-3-kinase (PI3K) /Akt signaling pathway (Han et al., 2012). Tao’s study also demonstrated that PI3K inhibitor LY294002 counteracted all the effects of puerarin. Their findings suggest that puerarin protects the hippocampus from IRI by activating the PI3K/Akt1/GSK-3β/MCL-1 signaling pathway (Tao et al., 2017).

### Inhibition of inflammatory response

Experiments by Lou’s team demonstrated that puerarin could inhibit neutrophil-mediated inflammatory response after brain I/R in rats. This effect may be mediated by the down-regulation of intercellular cell adhesion molecule-1 (ICAM-1) and NF-κB activity (Lou et al., 2004). In cerebral I/R, Chen also found that puerarin can protect the brain by decreasing the degradation of inhibitory protein κB (IP-κB), the activity of NF-κB, the expression of TNF-α mRNA, and the inflammatory reaction (Chen et al., 2005). Liu et al. found that pre-treatment with puerarin (intravenous injection) attenuated the inflammatory response in rats, which is associated with the activation of Janus-Activated kinase 2 (JAK2), signal transducers, transcriptional activator 3 (STAT3) and inhibition of NF-κB. These observations were inhibited by the α 7 nicotinic acetylcholine receptor (α 7nAchR) antagonist α-bungarotoxin (α-BGT). In addition, puerarin pre-treatment increased the expression of α 7nAchR mRNA in ischemic cerebral tissue. These data demonstrate that puerarin pre-treatment vigorously protects the brain against cerebral IRI and puerarin may play an anti-inflammatory role by activating the cholinergic anti-inflammatory pathway. (Liu et al., 2013). Zhou found that puerarin significantly improved neurological deficit, reduced infarct size and brain water content, and notably diminished the expression of Toll-like receptor-4 (TLR4), myeloid differentiation factor 88, NF-κB and tumor necrosis factor-a in the ischemic region (Zhou F. et al., 2014). These data indicated that puerarin exerts an anti-inflammatory protective effect on brain tissue with IRI by down-regulating the expression of multiple inflammatory factors (Zhou Y. X. et al., 2014). A recent study by Ling showed that due to the inhibition of NF-κB, TNF-α, IL-1β, IL-6 and the combination of salvianolic acid B (Sal-B), puerarin exerted a much stronger neuroprotective effect than Sal-B or puerarin alone, which provides a potential new drug and has great significance for the treatment of IS(Ling et al., 2018).

### Inhibition of autophagy response

In recent years, studies on the mechanism of puerarin injury to cerebral I/R have mainly focused on autophagy and its signaling pathways. His double immunofluorescence experiment showed that puerarin treatment markedly attenuated neuronal autophagy (He et al., 2017). In contrast, autophagy in astrocytes was only slightly attenuating, suggesting that puerarin may provide neuroprotection against cerebral ischemia. This biological function is associated with attenuating autophagy in neurons but not in astrocytes (He et al., 2017). Wang believes that puerarin relieves autophagy by activating the APMK-mTOR-ULK1 signaling pathway, and in subsequent studies proved that puerarin may reduce brain IRI by inhibiting autophagy via the AMPK-mTOR-Ulk1 signaling pathway (Wang et al., 2018; Huang et al., 2019). Cheng’s experiments demonstrated that the mechanism might be that puerarin reduces the expression of C-Jun N-terminal kinase (JNK), phosphorylates C-Jun N-terminal kinase (P-JNK), and then increases Bcl2, and interferes with the function of Beclin1 in autophagy (Cheng et al., 2019). Although some mechanisms have not been fully understood, puerarin plays an essential role in treating cerebral IRI in previous studies (Figure 3).

## Protective effect on spinal cord ischemia

The neuroprotective mechanism of puerarin against spinal IRI involves the transcriptional up-regulation of thioredoxin (Trx) mRNA and the reduction of apoptosis, and the mechanism may be related to antioxidant damage (Chen et al., 2020; Sang et al., 2004; Tian et al., 2015; Tian et al., 2011, 2013).

Initially, Sang et al.investigated the effect of puerarin on neural function and the histopathological changes after ischemic spinal cord injury in rabbits (Sang et al., 2004). They found that puerarin significantly improved neurological function and histopathological damage after transient spinal cord ischemia in rabbits (Sang et al., 2004). After a while, Tian’s study was to explore the optimal therapeutic timing and mechanism of puerarin treatment of spinal cord IRI (Tian et al., 2011). Their findings suggest that the neuroprotective effects of puerarin involve increased transcription of thioredoxin and decreased apoptosis (Tian et al., 2013). They later also confirmed by studies in mice that the neuroprotective mechanism of puerarin involved a decrease in glutamate release and mGluRs-1 mRNA expression and reduction of spinal injury was associated with inhibition of Cdk5 and p25. That inhibition of Cdk5 and p25 was one of the neuroprotective mechanisms in the puerarin treatment of acute I/R induced spinal injury in rats (Tian et al., 2015).

Since spinal cord injury involves inflammation and apoptosis of neurons, it is difficult to cure with systemic medication, so using advanced drug delivery techniques to administer natural active compounds (resveratrol and puerarin) can improve the patient’s condition. Through the latest research, Chen and his team found that nanoparticles loaded with resveratrol and puerarin can reduce free radicals produced by reperfusion injury-induced rats and reduce oxidative stress caused by IRI. Resveratrol and puerarin nanoparticles reduce glutathione (GSH), SOD and catalase (CAT) antioxidant levels, contributing to the improved overall health of patients (Chen et al., 2020). Various suggest that puerarin can protect the spinal cord from IRI by improving neural function and antioxidation.

## Protective effect on lung ischemia

In recent years, new medical methods have been continuously established and developed, such as pulmonary artery sleeve resection, lung transplantation, combined heart-lung transplantation, and pulmonary thrombolytic therapy, but pulmonary ischemia-reperfusion injury (PIRI) is always an essential factor affecting thrombolysis and prognosis after transplantation (Chen and Date, 2015; Laubach and Sharma, 2016; Sharma et al., 2018; Almeida et al., 2020). PIRI is a rapid and complex inflammatory response involving endothelial and epithelial injury/dysfunction, release of cytokines and damage-associated molecular patterns (DAMPs), and activate innate immune responses including alveolar macrophages, invariant natural Activation of killer T (iNKT) cells and neutrophils. Most of these responses are triggered by rapid and robust ROS generation, leading to cell/tissue damage, activation of multiple cell types, lipid membrane peroxidation, and secretion of inflammatory cytokines and DAMPs(Yellon and Hausenloy, 2007; Bonservizi et al., 2014; Ferrari and Andrade, 2015; Laubach and Sharma, 2016). Therefore, it is of great clinical significance to find a method to antagonize PIRI and strengthen the study of lung protection. Puerarin has been found to crush PIRI through different pathways and exert a protective effect on PIRI (Chen et al., 2004; Chen et al., 2010; Wang et al., 2015; Zheng et al., 2015).

Chen experimentally studied the effect of puerarin on reperfusion injury after thrombolytic therapy for acute pulmonary thromboembolism in Japanese rabbits and found that the Chinese herb puerarin can protect the lung against reperfusion injury after thrombolytic treatment in acute pulmonary thromboembolism which may be associated with antioxidation action (Chen et al., 2004). Then Zheng et al. studied the effect of puerarin on Fas/FasL mRNA expression in rabbit PIRI. The unilateral lung I/R model was used. Results: The abnormal changes in lung tissue morphology in each group were significantly less than those in the control group. Puerarin protects the lung from pii invasion by inhibiting the expression of Fas/FasL mRNA in lung tissue and reducing lung cell apoptosis (Zheng et al., 2015). Similarly, to investigate the effect of puerarin on expression of Fas/FasL protein during PIRI in the rabbits, experiments by Chen have also demonstrated that puerarin has notable protective effects on PIRI in rabbits by inhibiting Fas/FasL protein expression in lung tissue and decreasing apoptosis (Chen et al., 2010).

In search of more effective treatments, Wang and his team aimed to study the combined use of puerarin and edaravone on inhalation lung injury caused by black gunpowder smoke. A significant protective effect against neutrophil infiltration and tissue damage was observed. Myeloperoxidase activity and histopathological analysis were indicated. A combination of edaravone and puerarin is expected to be a new treatment option for patients with acute lung injury/acute respiratory distress syndrome (Wang et al., 2015). Therefore, puerarin can alleviate PIPI by alleviating endothelial and epithelial damage, inhibiting inflammatory response, inhibiting apoptosis, slowing down the innate immune response and inhibiting reactive oxygen species.

## Protective effect on intestinal ischemia

Intestinal IRI is one of the early pathological processes after severe trauma, major surgery, burn, infection and other stimuli. Intestinal IRI has a high incidence in clinical practice and often causes many serious complications. Intestinal IRI is also one of the critical factors for the occurrence and development of multiple organ failures after injury. Studies have shown that puerarin can effectively protect intestinal IRI through specific mechanisms (Liu and Chen, 2005; Tian et al., 2018).

Initially, Liu et al. reported the protective effect of puerarin against intestinal IRI (Liu and Chen, 2005). To explore its underlying mechanism in-depth, Tian and his team found that puerarin effectively ameliorated intestinal IRI by reducing histopathological changes. Puerarin significantly inhibited the expression of p66Shc and further attenuated intestinal ROS and epithelial cell apoptosis. Overexpression of p66Shc inhibited puerarin-induced protection as demonstrated by ROS accumulation and massive epithelial cell apoptosis *in vitro*. In addition, puerarin attenuated systemic injury, as evidenced by the reduced release of inflammatory cytokines, attenuated distal lung injury and improved survival. This finding may lead to therapeutic intervention for intestinal IRI (Tian et al., 2018).

## Prospects and conclusion

In summary, puerarin has an extensive range of pharmacological effects and has a significant protective effect on IRI in the heart, brain, spinal cord, lung, intestine and other organs (Table1). It is commonly used in ischemic cardiovascular and cerebrovascular diseases in clinical practice, with obvious advantages in clinical application. With the deepening of experimental pharmacological research and clinical research, puerarin is expected to be an effective drug for the clinical treatment of IRI.

**TABLE 1:** Protective mechanism of puerarin in different organs after ischemia-reperfusion injury.

Type of disease or condition	Pathway influenced by puerarin	Mechanism	References
Myocardial IRI	Cellular metabolism	Decrease lactate acid production and oxygen consumption	Fan et al. (1992a); Fan et al. (1992b)
	Inflammatory response	Inhibit TNF-α and IL-6 secretion	Zhu et al. (2001)
		Reduce neutrophil infiltration and oxidative stress	Liu et al. (2015); Wenjun et al. (2015)
	Ion channel and signal transduction	Open the mitochondrial ATP-sensitive potassium channel or the calcium-activated potassium channel and activate PKC	Gao et al. (2005); Gao et al. (2006); Gao et al. (2007); Pan et al. (2006); Tang et al. (2014)
	Angiogenesis	Induce VEGF, HIF-α, eNOS expression and accelerate angiogenesis	Ai et al. (2015); Zhang et al. (2006)
	Autophagy	Promote BAG3 expression and stimulate autophagy	Ma et al. (2016)
		Inhibit autophagy via the Akt signaling pathway	Tang et al. (2017)
Cerebral IRI	Apoptosis	Inhibit apoptosis through inhibiting Fas and P53 expression and up-regulating Bcl-2 expression	Cao et al. (2003); Wu et al. (2009); Yang et al. (2006)
	Reduce apoptosis by activating PI3K/Akt signaling pathway	Han et al. (2012); Tao et al. (2017)
	Inflammatory response	Inhibit multiple inflammatory factors expression such as ICAM-1, NF-κB, MYD88 and TNF-α	Chen et al. (2005); Ling et al. (2018); Lou et al. (2004); Zhou et al. (2014a)
		Activate cholinergic anti-inflammatory pathway	Liu et al. (2013)
	Autophagy	Alleviate autophagy by activating the APMK-mTOR-ULK1 signaling pathway	He et al. (2017); Huang et al. (2019); Wang et al. (2018)
		Decrease JNK and p-JNK expression, increase Bcl2 and interfere with the functions of Beclin1	Cheng et al. (2019)
Spinal cord IRI	Inflammatory response	Decrease oxidative stress by increasing thioredoxin transcription and decreasing free radicals	Chen et al. (2020); Tian et al. (2015); Tian et al. (2011); Tian et al. (2013)
	Apoptosis	Reduce neural cell apoptosis	Tian et al. (2015); Tian et al. (2011); Tian et al. (2013)
Lung IRI	Apoptosis	Inhibit Fas/FasL expression and decrease apoptosis	Chen et al. (2010); Zheng et al. (2015)
	Inflammatory response	Reduce neutrophil infiltration and oxidative stress	Chen et al. (2004); Wang et al. (2015)
Intestinal IRI	Inflammatory response	Inhibit p66Shc expression and attenuate intestinal ROS	Liu and Chen, (2005); Tian et al. (2018)
	Apoptosis	Inhibit p66Shc expression and attenuate apoptosis	Liu and Chen, (2005); Tian et al. (2018)

Note: IRI, ischemia-reperfusion injury; TNF-α, tumor necrosis factor α; IL-6, interleukin 6; ATP, adenosine triphosphate; PKC, protein kinase C; VEGF, vascular endothelial growth factor; HIF-α, hypoxia-inducible factor 1 α; eNOS, endothelial nitric oxide synthase; Bcl-2, B-cell lymphoma-2; BAG3, Bcl-2 associated athanogene 3; NF-κB, nuclear factor kappa-B; PI3K, phosphatidylin-ositol 3-kinase; ICAM-1, intercellular cell adhesion molecule-1; APMK, adenosine 5′-monophosphate (AMP)-activated protein kinase; mTOR, mammalian target of rapamycin; ULK1, The Unc-51 like autophagy activating kinase 1; JNK, c-Jun N-terminal kinase; ROS, reactive oxygen species.

Although these studies on the protective effects of puerarin in organ I/R are relatively preliminary, the evidence of its beneficial effects is encouraging. For example, there is evidence that puerarin plays a vital role in oxidative stress and inflammation in MI. However, there is still a need for further studies, particularly randomized controlled trials. First, with changes in the environment, diet and habits, these diseases are changing, so we don’t know if puerarin will still be suitable for these diseases. The second, the safety of puerarin, especially for long-term use, remains to be determined.

Finally, the route of administration in most studies was oral or gavage. Therefore, the exacting route and administration time also need to be determined. The development of puerarin derivatives is a hot topic, attracting many scholars worldwide (Zhang, 2019; Li et al., 2020; Pan et al., 2020; Li et al., 2021; Qin et al., 2021). There is no doubt that more effective puerarin preparations will be developed in the future to prevent and treat organ IRI.
